# 

**Published:** 1911-07-29

**Authors:** 


### BOOKS RECEIVED.

D. Appleton and Co.

"The Prevention of Infectious Diseases." By Alva H. Doty, M.D.

J. Murray.

"The Reduction of Domestic Mosquitos." By E. H. Ross.

Cassell and Co.

"The Students' Handbook of Surgical Operations." By Sir F. Treves, Bart., and Jonathan Hutchinson, F.R.C.S.

